# Case of Ossification in the Heart
†The Editors having mentioned Dr. Ashburner's communication to Mr. Rose, he was obliging enough to send the above case of very extensive ossification of the heart, which is preserved in the museum of the College of Surgeons, but without its history having been recorded until now. It strikingly illustrates the great extent to which this change of structure is capable of taking place, without bringing on any symptoms by which its existence can be detected before death.—Editors.


**Published:** 1822-12

**Authors:** Tho. Rose


ART. III.—*Case of Ossification in the Heart.*† By THO. ROSE, Esq.

RACHEL COOPER, sixty-nine years of age, died in the St. James's Infirmary, on the 9th of December, 1817. She was not subject to any particular complaints, but was feeble, and had long been afflicted with a large ulcer of the right leg, which had obliged her, during the last year of her life, to confine herself entirely to bed. The illness which proved fatal to her was dysentery. During the progress of it, she often complained of a

† The Editors having mentioned Dr. ASHBURNER's communication to Mr. ROSE, he was obliging enough to send the above case of very extensive ossification of the heart, which is preserved in the museum of the College of Surgeons, but without its history having been recorded until now. It strikingly illustrates the great extent to which this change of structure is capable of taking place, without bringing on any symptoms by which its existence can be detected before death.—EDITORS.

great sense of oppression about her heart. Her pulse was quick, but never was observed to intermit; and, as the dysenteric symptoms were extremely violent, all her complaints were referred to them. For several days before she died, the pulse was not perceptible at her wrist, and could hardly be felt even high in her arm.

I examined the body the day after her death. There was extensive ulceration of the inner coat of the great intestines through the whole of their course, especially of the lower part of the colon and of the rectum. There was a slight degree of recent inflammation of the pleura on both sides of the thorax, and that membrane adhered every where to the pericardium. The cavity of the pericardium was entirely obliterated. On the anterior part of the right auricle of the heart, the parietes of that cavity, to the extent of about two and a half square inches, were converted into bone. The ossification appeared to have begun in the pericardium, and to have extended itself gradually into the muscular structure; at least, it was more complete on the surface than towards the cavity of the auricle. A deposition of bone, to a still greater degree, had taken place in the muscular structure of the left ventricle, almost the whole of which, with the exception of a small space near the apex, formed a solid mass of bone. One segment of the valvulæ mitrales, between the left auricle and ventricle, was ossified, and formed a part of the boney mass. The semilunar valves of the aorta were but slightly ossified; the corpora sesamoidea alone being converted into bone. Bone was deposited in the coats of the aorta, in different parts of its course, especially round the origin of the three vessels sent off from its arch, and round that of the azygos arteries.

The heart has been preserved in the museum of the college in Lincoln's-Inn Fields.

*Park-place, St. James's-street; October* 1822.

